# Escore de Cálcio, Doença Coronária não Obstrutiva e Eventos Cardiovasculares

**DOI:** 10.36660/abc.20260405

**Published:** 2026-06-19

**Authors:** Marcio Hiroshi Miname

**Affiliations:** 1 Universidade de São Paulo Instituto do Coração do Hospital das Clínicas Faculdade de Medicina São Paulo SP Brasil Instituto do Coração do Hospital das Clínicas da Faculdade de Medicina da Universidade de São Paulo, São Paulo, SP – Brasil

**Keywords:** Aterosclerose, Cálcio, Doença da Artéria Coronariana

A doença cardiovascular é a principal causa de morbimortalidade no Brasil, com exceção de 2021, quando a pandemia de Covid-19 fez com que a doença infecciosa assumisse essa posição.^1^ A estratificação de risco cardiovascular para eventos ateroscleróticos tem o objetivo de identificar os indivíduos com maior risco e, consequentemente, aqueles com maior potencial de benefício da terapia farmacológica para redução desses eventos.^2^ Entretanto, as equações preditoras de risco ainda não apresentam calibração e discriminação suficientemente adequadas para a população brasileira,^3^ o que abre um precedente para incorporação de outros biomarcadores laboratoriais ou de imagem para que possam complementar e otimizar a precisão na estratificação de risco cardiovascular. O escore de cálcio coronário (CAC) foi amplamente analisado em diferentes populações, e sua associação com evento coronário aterosclerótico é robusta, culminando com sua incorporação nas diretrizes de dislipidemia brasileira e americana.^2,4^

Porém, deve-se ressaltar que o CAC reflete apenas a parte calcificada da placa de ateroma, não permitindo a avaliação do grau de estenose luminal, da composição total da placa ou da carga total aterosclerótica. Contudo, existe uma correlação entre maior CAC e maior carga de placa.

Dados do estudo Prospect demonstraram que a maioria dos eventos futuros se originou de placas não obstrutivas, com estenose média basal de apenas 32%.^5^ Apesar do aspecto discreto na angiografia, as lesões associadas a eventos apresentavam, ao ultrassom intravascular, maior carga de placa (≥70%), área luminal mínima ≤4 mm² e presença de fibroateroma de capa fina, características associadas independentemente a maior risco de eventos.^5^

A relação entre placas ateroscleróticas não obstrutivas e CAC foi abordada no artigo Escore de Cálcio das Artérias Coronárias, Fatores de Risco e Desfechos Clínicos na Doença Arterial Coronariana Não Obstrutiva: Um Estudo de Seguimento de Longo Prazo.^6^ Os autores avaliaram 2509 indivíduos submetidos à angiotomografia de coronárias por dor torácica e sem presença de placa obstrutiva (acima de 50%). Os pacientes foram acompanhados para avaliação de eventos cardiovasculares, e também foram levantados os resultados de uma segunda angiotomografia de coronárias, realizada em 396 pacientes a critério do médico assistente. Cerca de 76% dos pacientes não apresentavam placa ou tinham carga aterosclerótica leve. Quase metade dos pacientes (45%) apresentava CAC=0, enquanto um quinto apresentava CAC ≥100. Os autores definiram a carga de placa com base em um critério que considerou o número de lesões e grau de obstrução. Houve uma correlação entre o CAC e a carga de placa: em pacientes com CAC zero, predominou a ausência de lesões, e se observou aumento da carga aterosclerótica com a elevação do CAC.

Em relação aos pacientes que realizaram uma segunda angiotomografia, a média de intervalo de tempo entre os exames foi de 6,5 anos, e a análise multivariada revelou que sexo masculino e idade foram os preditores da progressão do CAC. Ocorreram 123 eventos cardiovasculares (4,9%) durante média de seguimento de 8,9 anos. A análise de regressão logística multivariada mostrou que idade >65 anos, sedentarismo, sexo masculino e história familiar de doença arterial coronariana (DAC) foram associados com eventos cardiovasculares, porém não houve associação com CAC quando este foi analisado por categorias. Entretanto, um outro modelo, no qual os fatores de risco foram apresentados como número total de fatores presentes em cada indivíduo, tanto esse parâmetro quanto as categorias de CAC se associaram a eventos cardiovasculares. Dessa forma, os autores concluem que, em pacientes sem DAC obstrutiva, o CAC se correlacionou com carga de placa e fatores de risco.

O artigo^6^ abre um precedente para discussão da associação entre CAC e placas ateroscleróticas não obstrutivas.^6^ A importância das placas não obstrutivas foi avaliada no estudo Prospect II, que incluiu pacientes com infarto do miocárdio recente. Nesse estudo, a maioria dos eventos durante quatro anos de seguimento se originou de placas não obstrutivas presentes no momento do evento índice.^7^ As lesões com maior probabilidade de causar futuros eventos cardiovasculares maiores apresentavam alto conteúdo lipídico e grande carga de placa.^7^ Ao demonstrar associação entre CAC e carga de placa em indivíduos sem DAC obstrutiva, o estudo^6^ reforça o papel do CAC na avaliação de risco desses indivíduos.

Outro ponto de destaque é a análise de progressão do CAC realizada em um subgrupo da casuística, no qual cerca de 41% dos indivíduos apresentaram progressão. Porém, cabe ressaltar que, no baseline 68% dos pacientes faziam uso de hipolipemiantes. Evidências prévias mostram associação entre estatinas e aumento na densidade da calcificação da placa de ateroma.^8^ Além disso, estudo prévio mostra interação entre CAC, uso de estatina e eventos cardiovasculares.^9^ Portanto, esse achado do estudo deve ser analisado sob a ótica da terapia com estatina.

O risco de eventos associados à doença aterosclerótica atualmente não deve ser entendido como algo exclusivamente relacionado à presença da placa obstrutiva, mas também ao fenótipo de placa, sua interação com fatores de risco e até mesmo fatores inflamatórios sistêmicos e locais da parede vascular. Este último pode ser avaliado pelo índice de atenuação da gordura pericoronária (FAI). De fato, o FAI é um importante marcador de eventos em pacientes com placas não obstrutivas, como elegantemente demonstrado no estudo ORFAN.^10^ O artigo^6^ agrega neste campo de conhecimento, e os autores devem ser congratulados pela publicação.
