# Supplementary material for: Escore de Cálcio, Doença Coronária não Obstrutiva e Eventos Cardiovasculares
Source: Arq Bras Cardiol. 2026 Jun 19;123(5):e20260405. [Article in Portuguese] doi: 10.36660/abc.20260405 (PMC13399061; doi:10.36660/abc.20260405)
Supplement: Translation

# Translation: Calcium Score, Non-Obstructive Coronary Artery Disease and Cardiovascular Events

**DOI:** 10.36660/abc.20260405i

**Authors:** Marcio Hiroshi Miname

**Affiliations:** 1 São Paulo SP Brazil Instituto do Coração do Hospital das Clínicas da Faculdade de Medicina da Universidade de São Paulo, São Paulo, SP – Brazil

**Keywords:** Atherosclerosis, Calcium, Coronary Artery Disease

Cardiovascular disease is the leading cause of morbidity and mortality in Brazil, except for 2021, when the COVID-19 pandemic caused the infectious disease to take that position.^1^ Cardiovascular risk stratification for atherosclerotic events aims to identify individuals at higher risk and, consequently, those with the greatest potential benefit from pharmacological therapy to reduce these events.^2^ However, risk prediction equations still lack sufficient calibration and discrimination for the Brazilian population.^3^ This opens a precedent for the incorporation of other laboratory or imaging biomarkers to complement and optimize the accuracy of cardiovascular risk stratification. The coronary calcium score (CCS) has been extensively analyzed in different populations, and its association with atherosclerotic coronary events is robust, culminating in its incorporation into Brazilian and American dyslipidemia guidelines.^2.4^

However, it should be noted that the CCS only reflects the calcified part of the atheromatous plaque, not allowing for the assessment of the degree of luminal stenosis, the total composition of the plaque, or the total atherosclerotic burden. Nevertheless, there is a correlation between higher CCS and higher plaque burden.

Data from the Prospect study demonstrated that most future events originated from non-obstructive plaques, with a mean baseline stenosis of only 32%.^5^ Despite their subtle appearance on angiography, lesions associated with events showed, on intravascular ultrasound, a higher plaque burden (≥70%), a minimum luminal area ≤4 mm², and the presence of thin-cap fibroatheroma, characteristics independently associated with a higher risk of events.^5^

The relationship between non-obstructive atherosclerotic plaques and CCS was discussed in the article Coronary Artery Calcium Score, Risk Factors, and Clinical Outcomes in Nonobstructive Coronary Artery Disease: A Long-Term Follow-Up Study.^6^ The authors evaluated 2509 individuals who underwent coronary angiotomography for chest pain, without the presence of obstructive plaque (above 50%). Patients were followed up for cardiovascular event assessment, and the results of a second coronary angiotomography, performed in 396 patients at the discretion of the attending physician, were also collected. Approximately 76% of patients had no plaque or had a mild atherosclerotic burden. Almost half of the patients (45%) had a CCS of 0, while one-fifth had a CCS ≥100. The authors defined plaque burden based on a criterion that considered the number of lesions and the degree of obstruction. There was a correlation between CCS and plaque burden: in patients with zero CCS, the absence of lesions predominated, and an increase in atherosclerotic burden was observed with an increase in CCS.

Regarding patients who underwent a second coronary angiotomography, the average time interval between examinations was 6.5 years, and multivariate analysis revealed that male sex and age were predictors of CCS progression. One hundred and twenty-three cardiovascular events (4.9%) occurred during an average follow-up of 8.9 years. Multivariate logistic regression analysis showed that age >65 years, sedentary lifestyle, male sex, and family history of coronary artery disease (CAD) were associated with cardiovascular events, but there was no association with CCS when analyzed by categories. However, another model, in which risk factors were presented as the total number of factors present in each individual, showed that both this parameter and CCS categories were associated with cardiovascular events. Thus, the authors conclude that, in patients without obstructive CAD, CCS correlated with plaque burden and risk factors.

The paper^6^ opens a precedent for discussion of the association between CAD and non-obstructive atherosclerotic plaques.^6^ The importance of non-obstructive plaques was evaluated in the Prospect II study, which included patients with recent myocardial infarction. In this study, most events during the four years of follow-up originated from non-obstructive plaques present at the time of the index event.^7^ The lesions most likely to cause future major cardiovascular events had a high lipid content and a large plaque burden.^7^ By demonstrating an association between CAD and plaque burden in individuals without obstructive CAD, the study^6^ reinforces the role of the CCS in assessing the risk of these individuals.

Another highlight is the analysis of CCS progression performed in a subgroup of the case series, in which approximately 41% of individuals showed progression. However, it is worth noting that at baseline, 68% of patients were using lipid-lowering drugs. Previous evidence shows an association between statins and increased calcification density of atherosclerotic plaque.^8^ Furthermore, a previous study shows an interaction between CCS, statin use, and cardiovascular events.^9^ Therefore, this finding from the study should be analyzed from the perspective of statin therapy.

The risk of events associated with atherosclerotic disease should not be understood as exclusively related to the presence of obstructive plaque, but also to plaque phenotype, its interaction with risk factors, and even systemic and local inflammatory factors of the vascular wall. The latter can be assessed by the pericoronary fat attenuation index (FAI). In fact, FAI is an important marker of events in patients with non-obstructive plaques, as elegantly demonstrated in the ORFAN study.^10^ The article^6^ contributes to this field of knowledge, and the authors should be congratulated on the publication.
